# Genome-Wide Copy Number Variation in Sporadic Amyotrophic Lateral Sclerosis in the Turkish Population: Deletion of EPHA3 Is a Possible Protective Factor

**DOI:** 10.1371/journal.pone.0072381

**Published:** 2013-08-26

**Authors:** Özgün Uyan, Özgür Ömür, Zeynep Sena Ağım, Aslıhan Özoğuz, Hong Li, Yeşim Parman, Feza Deymeer, Piraye Oflazer, Filiz Koç, Ersin Tan, Hilmi Özçelik, A. Nazlı Başak

**Affiliations:** 1 Suna and İnan Kıraç Foundation Neurodegeneration Research Laboratory, Molecular Biology and Genetics Department, Bogazici University, Istanbul, Turkey; 2 Samuel Lunenfeld Research Institute, Mount Sinai Hospital, Toronto, Canada; 3 Neurology Department, Istanbul Medical School, Istanbul University, Istanbul, Turkey; 4 Neurology Department, Medical School, Çukurova University, Adana, Turkey; 5 Neurology Department, Hacettepe University, Ankara, Turkey; 6 Department of Laboratory Medicine and Pathobiology, University of Toronto, Toronto, Canada; National Institutes of Health, United States of America

## Abstract

The genome-wide presence of copy number variations (CNVs), which was shown to affect the expression and function of genes, has been recently suggested to confer risk for various human disorders, including Amyotrophic Lateral Sclerosis (ALS). We have performed a genome-wide CNV analysis using PennCNV tool and 733K GWAS data of 117 Turkish ALS patients and 109 matched healthy controls. Case-control association analyses have implicated the presence of both common (>5%) and rare (<5%) CNVs in the Turkish population. In the framework of this study, we identified several common and rare loci that may have an impact on ALS pathogenesis. None of the CNVs associated has been implicated in ALS before, but some have been reported in different types of cancers and autism. The most significant associations were shown for 41 kb and 15 kb intergenic heterozygous deletions (Chr11: 50,545,009–50,586,426 and Chr19: 20,860,930–20,875,787) both contributing to increased risk for ALS. CNVs in coding regions of the MAP4K3, HLA-B, EPHA3 and DPYD genes were detected however, after validation by Log R Ratio (LRR) values and TaqMan CNV genotyping, only EPHA3 deletion remained as a potential protective factor for ALS (p = 0.0065024). Based on the knowledge that EPHA4 has been previously shown to rescue SOD1 transgenic mice from ALS phenotype and prolongs survival, EPHA3 may be a promising candidate for therepuetic interventions.

## Introduction

Amyotrophic lateral sclerosis (ALS) is a complex neurodegenerative disorder impacted by genetic and environmental factors. The disease presents as familial ALS (fALS) in 10% of patients, whereas the remaining 90% represents the non-familial form, called sporadic ALS (sALS) [1]. The genome-wide presence of copy number variations (CNVs), which was shown to affect the expression and function of genes, has been recently suggested to confer risk for various human disorders, including ALS. To date, using high-density genome-wide single nucleotide polymorphism (SNP) data, four studies restricted to European and US-European ALS populations investigated the presence of CNVs. Cronin *et* *al.* identified several candidates, including the deletion in C14orf177, and deletion and duplication of the GSDMDC1 and STS genes among 408 Irish and 868 Dutch individuals, respectively [2]. Blauw *et* *al.* investigated 406 patients with sporadic ALS versus 404 controls and identified several candidates, such as deletion of the CLEC3A and WWOX genes [3]. Wain *et* *al.* analyzed 730 ALS cases and 789 controls to find CNVs associated with ALS. They reported several intergenic and gene loci, including two top coding candidates, e.g. duplications of the RDH13 and FBXL2 genes [4]. Blauw et *al*., conducted a genome-wide screen of 1,875 cases and 8,731 controls (including over 8,000 individuals in replication set), this study revealed deletions and duplications of DPP6 and deletions of NIPA1 loci to be candidates for ALS development [5].

In a recent study, duplication of the SMN1 gene, responsible for spinal muscular atrophy, has been associated with sALS [6]; furthermore, homozygous SMN2 deletions were found to be protective in the Swedish population [7]. In this study, we have investigated for the first time the presence of CNVs in a Turkish ALS cohort with matched healthy controls; we were able to identify several candidates that may impact the development of ALS in the Turkish population.

## Materials and Methods

### Ethics Statement

Written informed consent was taken from all patients involved in this study. Written consent for affected children/minors was obtained from their parents. The approval of the use of patient samples was obtained from the Ethics Committee of Bogaziçi University, Istanbul. Control samples were collected anonymously from the Microbiology Department of Haydarpasa State Hospital, Istanbul. The control samples used in this study were described in a previous publication [8].

### Study Population and Pre-CNV Analysis

Sporadic ALS patients were referred to our center from different hospitals and neurology clinics throughout Turkey. El Escorial Criteria were applied for clinical diagnosis [9]. Genotyping, using the Illumina HumanOmniExpress 733K SNP array chip, was performed for 117 Turkish sALS patients and 109 ethnic, gender- and age-matched healthy controls. All samples had a high quality genotyping rate (genotype call >98%, total genotyping rate: 0.994). The mean age of onset was 47.8 (age range: 17–79) for cases and the mean age was 53.4 for controls (age range: 23–84). The gender proportion of male to female was 1.32 for cases and 1.4 for controls.

### CNV Analysis and Post-CNV Calling

PennCNV software was used for quality control and CNV analysis [10]. The raw GWAS SNP information, including Log R Ratio (LRR) and B allele frequencies (BAF) values were extracted for each individual. First, CNV calls were maintained with confidence scores using default parameters without any criteria yielding a total of 25,000 CNVs. We applied a set of filtering criteria (as recommended by the algorithm) to exclude poor quality samples and used the GC model signal adjustment to reduce false positive calls in individuals with high fluctuation of genomic signal waviness. After signal adjustment, individuals with >100 CNVs were excluded from analysis. To discard false positives, the generated CNV calls were also filtered using confidence value scores provided for each sample by PennCNV output. A confidence score of 10 or larger has been suggested as a threshold to classify reliable CNV calls [11]. After elimination of individuals and CNV calls with a low confidence value (conf<10), approximately 5,000 CNV calls were obtained and included in the final analysis.

### Defining the CNV Loci

CNV calls were grouped into loci having an intersection of at least 1 kb. The overlapping parts of CNV calls were defined as CNV loci. Each of these CNV loci represents all CNV calls that were present in that particular region. To identify CNV loci that are novel, we compared our results with those published in the Database of Genomic Variants (DGV) [12]. We used the gene annotation of the University of California Santa Cruz (UCSC) Genome Browser (http://genome.ucsc.edu/) [13] to identify genes that were located within or partially overlapped with CNV loci.

### Validating CNV Calls by Interpreting Log R Ratios (LRRs)

We additionally analyzed the top candidate regions, identified by the PennCNV tool to see whether PennCNV detected CNV calls properly. Individuals were categorized into two groups, controls without CNV versus patients and controls with CNV. The LRR values, obtained from SNP genotyping data, were extracted within the candidate CNV regions, as well as regions in the proximity of CNV loci. Average LRRs of SNPs in both groups were calculated and plotted for comparison. t-test and Mann-Whitney U test were applied to LRR value of each SNP in both groups for statistical significance. p-value of threshold is defined as p<0.0005.

### Validation of CNV Calls Using Quantitative PCR

To validate the candidate CNVs in the MAP4K3, HLA-B and EPHA3 gene regions, TaqMan assays Hs02852136_cn, Hs03605931_cn and Hs03458738_cn were used for genotyping each locus, respectively. TaqMan Copy Number Reference Assay (genotyping human the RNase P gene) was used as reference. Experiments were performed according to the manufacturer’s instructions and StepOnePlus instrument (Applied Biosystems Inc., USA) was used for qPCR. For the CNV analysis, C_T_ values were obtained from StepOne Software v.2.2.2 (Applied Biosystems Inc., USA) and then imported to Copy Caller Software v.2.0 (Applied Biosystems Inc., USA) to determine copy numbers.

### Statistical Analysis

Fisher’s exact test was used to carry out the case-control association analysis of CNVs identified in the Turkish population. No multiple testing was applied. The significance threshold was chosen as p<0.05. For comparison of CNV sizes and mean numbers per individual between cases and controls, two-tailed Mann-Whitney U test was used.

## Results

### Genome-wide CNV Analysis

Analysis of the 733K GWAS markers using LRR and BAF values, yielded ∼25,000 CNV calls with default parameters. After the GC signal adjustment model, 18,200 CNV calls were generated. In addition, individuals with high number of CNV calls, and CNV calls with low confidence values were eliminated. As a result, two ALS patients and three healthy controls with more than ∼10,000 CNV calls were discarded. In addition, 3,000 calls were excluded from CNV analysis due to low confidence threshold (conf<10.00). After exclusion of CNV calls based on the above criteria, 4,935 CNVs in 115 ALS patients and 106 controls were considered for further investigation.

### Characteristics of the CNVs in Cases and Controls

Among 4,935 CNV calls detected, 2,736 were found in cases and 2,199 in controls. The average number of all types of CNVs per individual were significantly higher in ALS patients (23.8) compared to controls (20.7) (p = 0.002). In addition, the range of CNV lengths was also significantly higher in cases (87.1 kb) as compared to controls (85.2 kb) (p = 0.012) (Table 1).

**Table 1:** Summary Statistics of CNV screening in 115 ALS patients and 106 controls.

Categories	ALS patients	Control	p-value
Average CNV number per individual (Range)	23.8 (9–85)	20.7 (7–76)	**0.002**
Average CNV length (kb)/(Range of lengths)	87.1/(97 bp–8,438 kb)	85.2/(33 bp–3,538 kb)	**0.012**
Average SNP number per CNV (Range)	14.21 (3–311)	15.45 (3–322)	0.209

We have also investigated the type of CNVs (duplications, heterozygous and homozygous deletions) in cases and controls. Out of 2736 CNV calls in ALS patients, 1115 CNVs were duplications and the rest were deletions. In controls, 868 duplications and 1331 deletions were detected. Two-tailed Mann-Whitney U test has shown statistically significant association of mean duplication per individual in cases (9.78) compared to controls (8.19) (p = 0.001). Median size of heterozygous deletions were also found to be significant in ALS cases (27.39) as compared to controls (22.8) (p = 0.024) (Table 2).

**Table 2:** Characteristics of CNV calls.

Type of CNV		Cases	Controls	p value
Duplication				
	Total	1115	868	
	Mean per individual (Range)	9.78	8.19	**0.001**
	Median Size, kb (Range)	53.85 (0–1448)	53.52(0–869)	0.702
Heterozygous deletion				
	Total	1404	1099	
	Mean per individual (Range)	12.32	10.37	0.116
	Median Size, kb (Range)	27.39 (0–8438)	22.8 (0–3539)	**0.024**
Homozygous deletion				
	Total	217	232	
	Mean per individual (Range)	1.90	2.19	0.257
	Median Size, kb (Range)	4.69 (0–337)	3.87 (0–338)	0.956
	Total number of CNVs	2736	2199	

### Frequent CNVs

Case-control association analyses have implicated the presence of both common (frequency>5%) and rare (frequency<5%) CNVs in the Turkish population. Among the 20 statistically significant (p<0.05) common CNV loci located within intergenic and gene coding regions, two third represented single copy losses (heterozygous deletion) with an exception of a homozygous deletion, and one third copy number gains (Table 3). Out of 20 CNVs, six represented gains, whereas 14 were losses, including 13 heterozygous and one homozygous deletion. Six out of 20 CNVs had higher frequencies in controls, thus representing changes protective for ALS. The remaining 14 CNVs had low frequencies in controls (<2%), accounting for increased ALS risk. Among the 20, 18 were previously reported [14], [15], [16], [17] in DGV and two, MAP4K3 (mitogen-activated protein kinase kinase kinase kinase 3) and the intergenic locus (chr3: 84,486,776–84,510,027), were novel.

**Table 3:** Association results of CNVs observed in analysis with p<0.05 (Fisher’s Exact Test).

Chr	Start (bp)	End (bp)	Change	Novel/reported	ALS	%	Control	%	p value	Intergenic/Gene region	Gene Name
11	50,545,009	50,586,426	loss	r	21	18.42	2	1.89	2.94E-05	intergenic	
19	20,860,930	20,875,787	loss	r	15	13.16	2	1.89	0.001320	intergenic	
2	39,372,016	39,428,488	gain	n	14	12.28	2	1.89	0.0023976	MAP4K3	mitogen-activated protein kinase kinase kinase kinase 3
3	84,486,776	84,510,027	gain	n	11	9.65	1	0.94	0.0037285	intergenic	
6	31,389,749	31,393,270	loss*	r	8	7.02	0	0.00	0.0045992	HLA-B,HLA-B*0707	major histocompatibility complex, class I, B
5	151,496,845	151,499,002	loss	r	5	4.39	16	15.09	0.0061567	AK001582	
3	89,485,137	89,499,861	loss	r	2	1.75	11	10.38	0.0065024	EPHA3	EPH receptor A3
2	208,064,053	208,066,082	loss	r	2	1.75	11	10.38	0.0065024	intergenic	
1	97,830,032	97,841,389	gain	r	12	10.53	2	1.89	0.0076187	DPYD	dihydropyrimidine dehydrogenase
4	153,010,030	153,012,241	loss	r	12	10.53	2	1.89	0.0076187	intergenic	
2	89,731,562	89,757,456	loss	r	6	5.26	0	0.00	0.0181413	near centromeric region	
1	147456822	147655013	loss	r	17	14.91	6	5.66	0.0203443	NBPF20	neuroblastoma breakpoint family, member 20
7	61,792,309	61,797,361	loss	r	12	10.53	3	2.83	0.0208864	near centromeric region	
11	107,166,452	107,175,438	gain	r	8	7.02	1	0.94	0.022996	SLC35F2	solute carrier family 35, member F2
13	63,241,820	63,285,508	gain	r	8	7.02	1	0.94	0.022996	AK127969	uncharacterized protein FLJ25694
6	62,237,262	62,247,872	loss	r	13	11.40	4	3.77	0.0292191	intergenic	
3	190,847,117	190,849,456	loss	r	6	5.26	14	13.21	0.034200	TP63	tumor protein p63
12	58,222,193	58,228,389	loss	r	23	20.18	11	10.38	0.0333527	intergenic	
15	54,580,082	54,588,851	loss	r	1	0.88	6	5.66	0.0486939	intergenic	
8	47,647,579	47,654,762	gain	r	1	0.88	6	5.66	0.0486939	intergenic	

loss* Homozygous deletion.

As compiled in Table 3, the most significant association was shown for a 41 kb intergenic heterozygous deletion (chr11: 50,545,009–50,586,426) in the proximity of the centromere. This intergenic deletion was observed in 18.26% of patients and 1.89% of controls (p = 3.23×10^−5^), thus conferring approximately 10-fold increased risk for ALS. The second most significant CNV association was detected for another intergenic 15 kb heterozygous deletion (Chr19: 20,860,930–20,875,787). This previously reported deletion was observed in 13.16% of patients and 1.89% of controls, also conferring increased ALS risk (p = 0.00132) (Figure S1).

CNVs in coding regions of the MAP4K3, HLA-B (major histocompatibility complex, class I, B), EPHA3 (EPH receptor A3) and DPYD (dihydropyrimidine dehydrogenase) genes were also found to be associated with ALS risk orprotection. The novel 56,472 bp duplication, covering both exonic and intronic parts of MAP4K3 (chr2: 39,372,016–39,428,488), was observed in 12.17% of cases and in only 1.89% of controls, thus conferring significantly increased ALS risk (p = 0.0025). The second candidate, an 11.5 kb duplication in the coding region of DPYD, was also significantly associated with ALS (p = 0.008, detected in 10.5% of ALS patients and 2% of controls). In addition, a 4 kb homozygous deletion (chr6: 31,389,749–31,393,270) in the intronic part of the HLA-B gene was observed in 7% ALS patients and was absent in controls (p = 0.0046). As opposed to the above loci, a 14 kb heterozygous deletion, spanning both the exonic and intronic parts of EPHA3 (chr3: 89,485,137–89,499,861) was found to be significantly protective for ALS, present in ∼10% of controls and only 2% of ALS patients (p = 0.0062) (Table 3) (Figure S1). This locus was previously identified in several studies on healthy individuals [14], [15].

### Rare CNVs

Although statistically not significant, ∼15 rare CNV regions (with less than 5% frequency in cases or controls) were also detected in this study. Among these, eight represented gains, whereas seven were losses, including six heterozygous deletions and a homozygous intergenic deletion. Some of those were ALS-specific and others were control-specific. The top candidates in coding regions included the novel CNV loci in ACYP2 (acylphosphatase 2, muscle type), LPHN3 (latrophilin 3) and TAC1 (tachykinin, precursor 1) genes (Table 4). Besides rare CNVs, approximately 500 CNV calls were individual-specific (private).

**Table 4:** Rare CNVs observed in analysis.

Chr	Start (bp)	End (bp)	Change	Novel/reported	ALS	%	Control	%	Intergenic/Gene region	Gene Name
2	54,343,530	54,356,415	loss	n	4	3.51	0	0.00	ACYP2	acylphosphatase 2, muscle type
4	62,506,258	62,566,026	gain	n	4	3.51	0	0.00	LPHN3	latrophilin 3
6	19,153,539	19,156,752	loss*	r	4	3.51	0	0.00	intergenic	
8	2,798,648	2,815,064	gain	r	4	3.51	0	0.00	CSMD1	CUB and Sushi multiple domains 1
20	61,791,411	61,844,885	gain	r	4	3.51	0	0.00	RTEL1	regulator of telomere elongation helicase 1
1	116,197,766	116,202,917	loss	r	0	0.00	2	1.89	intergenic	
1	150,519,809	150,526,366	loss	n	0	0.00	4	3.77	intergenic	
3	150,447,382	150,450,025	loss	r	0	0.00	3	2.83	intergenic	
3	156,963,791	156,988,291	gain	r	0	0.00	3	2.83	C3orf33	chromosome 3 open reading frame 33
3	163,637,770	163,690,547	gain	n	0	0.00	5	4.72	intergenic	
7	97,203,867	97,226,966	gain	n	4	3.51	0	0.00	TAC1	tachykinin, precursor 1
8	70,595,842	70,622,357	gain	r	4	3.51	0	0.00	SULF1	sulfatase 1
9	71,289,938	71,305,531	loss	r	3	2.63	0	0.00	APBA1	amyloid beta (A4) precursor protein-binding, family A, member 1
9	138,380,284	138,416,305	gain	r	0	0.00	4	3.77	CARD9	caspase recruitment domain family, member 9
10	58,574,865	58,606,945	loss	r	0	0.00	4	3.77	intergenic	

loss* Homozygous deletion.

### CNV Call Validation Using LRR Values

**Figure 1:**
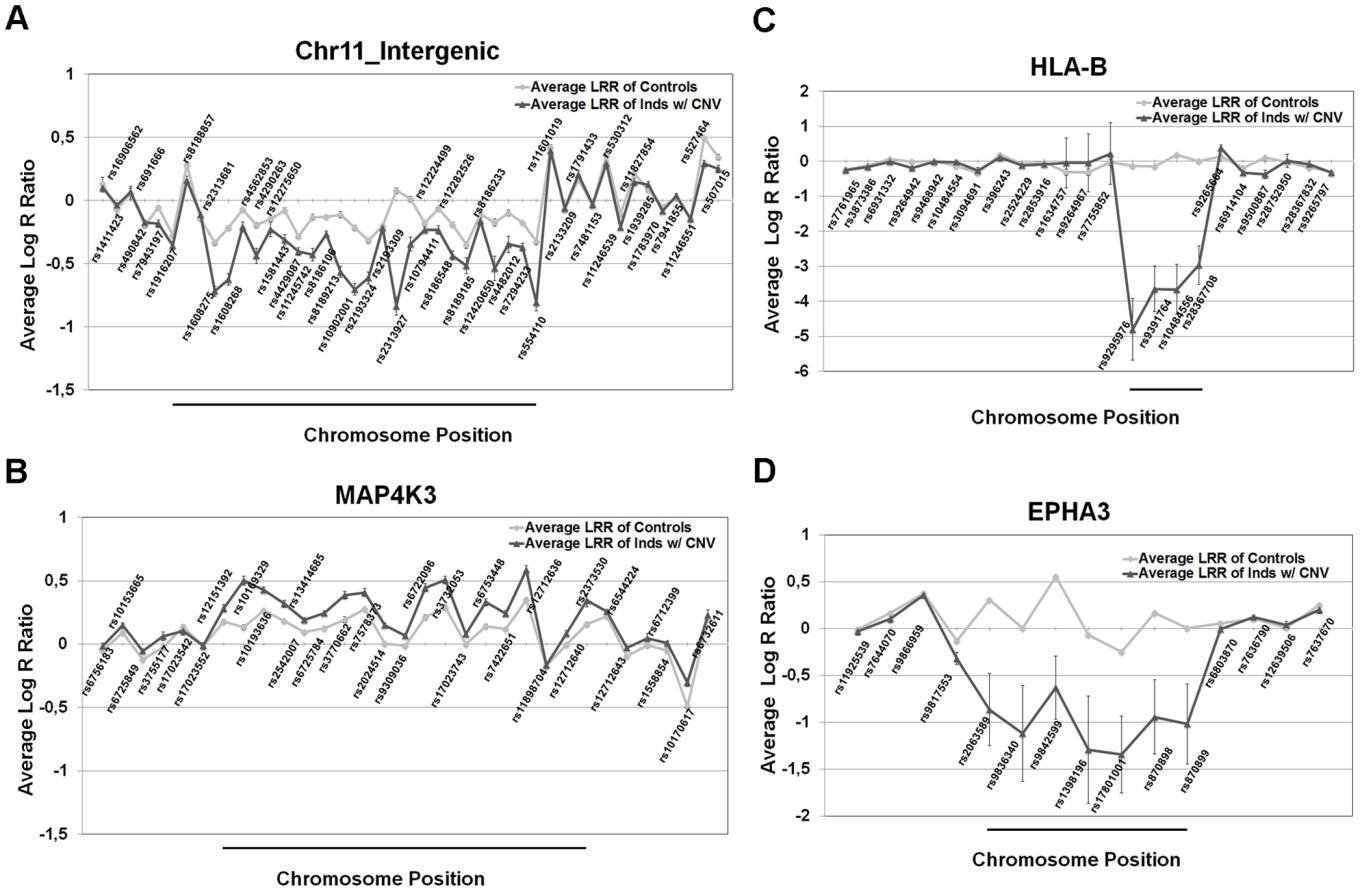
CNV call validation via LRR values. LRR values of controls and individuals with CNV for particular SNPs were extracted to check CNV calls of PennCNV tool. Average LRR values of each SNP for both groups were calculated and plotted. Consecutive SNPs in chromosome position line do not demonstrate the exact distance. The CNVs detected by PennCNV were shown by black bars. (a) Loss of an intergenic region on chromosome 11 between rs1916207 and rs554110 (chr11: 50,545,009–50,586,426), (b) gain of MAP4K3 gene between rs12151392 and rs2373530 (chr2: 39,372,016–39,428,488), (c) homozygous loss of HLA-B gene between rs9295976 and rs28367708 (chr6: 31,389,749–31,393,270), (d) loss of EPHA3 gene between rs2063589 and rs870899 (chr3: 89,485,137–89,499,861) (NCBI37/hg19).

Top significant CNV loci indicated by PennCNV were also investigated by plotting SNP data of all individuals (Figure S2). Average LRR values of SNPs in top candidate CNV loci were analyzed. In four distinct locations, changes in the intergenic region on chromosome 11, MAP4K3, HLA-B and EPHA3 genes including their proximity regions from both ends were found to be significant. The difference in LRRs of SNPs was observed in CNV regions of loss of intergenic region on chr11 and gain of MAP4K3 gene, loss of HLA-B and EPHA3 genes. Unlike CNV regions, upstream and downstream sequences of these regions did not show any significant change (Figure 1). Other top candidate CNV loci including intergenic loci, DPYD, NBPF20, SLC35F2 and TP63 were not found to be significant according to LRR validation.

**Figure 2:**
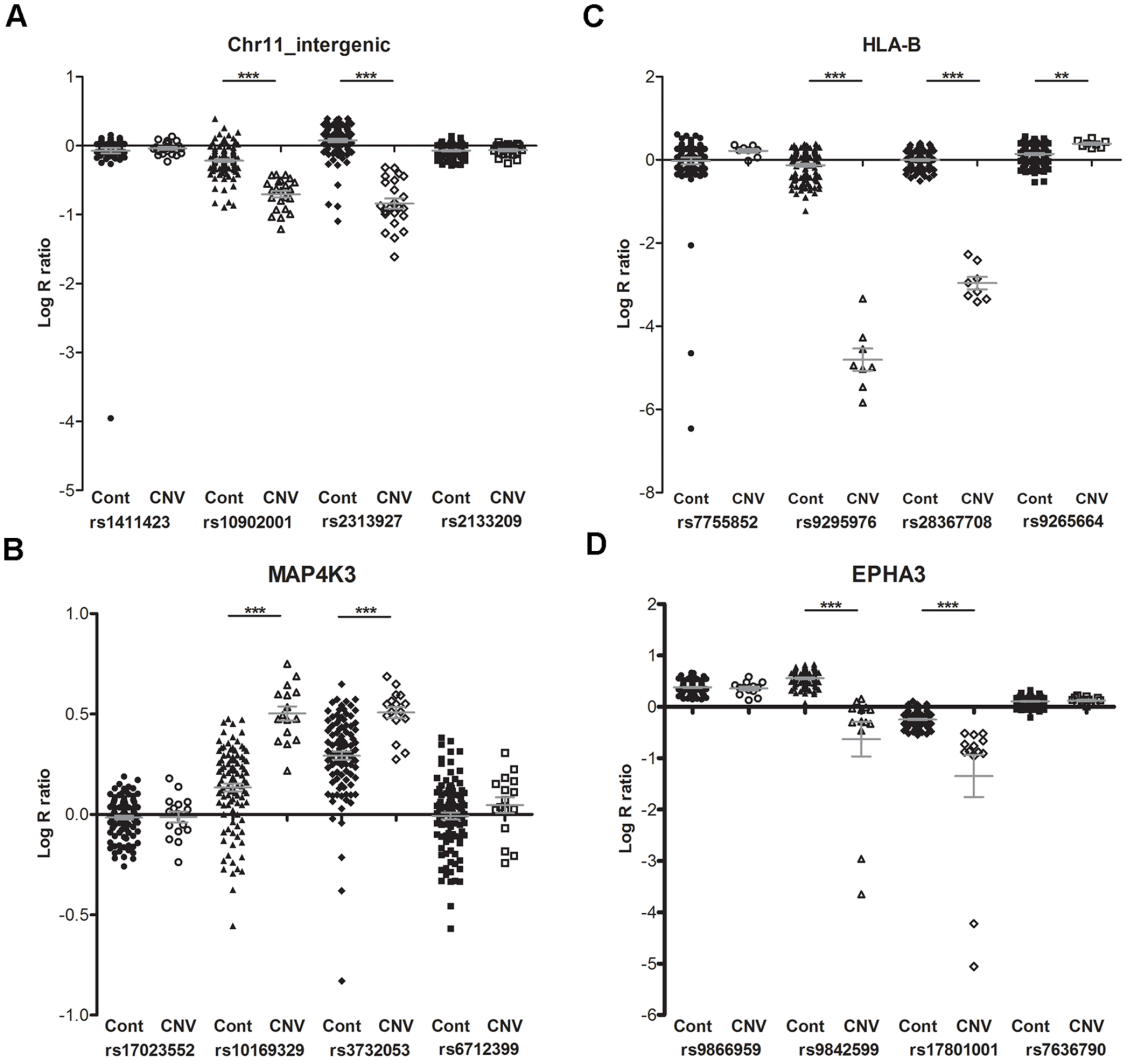
Distribution of LRR values of controls and individuals with CNV at SNP level. LRRs of all individuals were extracted and plotted. “*Cont*” indicates control individuals and “*CNV*” indicates individuals with a CNV. “*” indicates the significance level between controls and CNV. 4 SNPs, including one upstream, one downstream and two within the CNV region, were selected and LRRs were plotted for (a) intergenic reigon on chromosome 11, (b) MAP4K3 gene, (c) HLA-B gene and (d) EPHA3 gene.

To validate the CNV loci predicted, LRR values of these individuals (the intensities of controls and individuals with CNV, obtained from the array) at a particular SNP were analyzed for each CNV region, including chromosome 11, MAP4K3, HLA-B and EPHA3 genes (Figure 2). rs1411423 and rs2133209, located nearby the intergenic CNV region of chr11, did not show any intensity changes in any individual, whereas lower intensity changes were observed in individuals with CNV at rs10902001 and rs2313927 (located within the CNV loci) compared to controls. These differences were highly significant (p<0.0005) (Figure 2a). In MAP4K3 gene region, LRRs of SNPs (rs17023552 and rs6712399) nearby the CNV loci did not display any difference, on the other hand SNPs located within the CNV loci showed significant changes (p<0.0005). Higher intensities were observed in individuals with CNV indicating duplication (Figure 2b). In HLA-B region, very low insenties were obtained in individuals with CNV when compared to controls. Intensities of controls were normal at each SNP, however, lower intensities at rs9295975 and rs28367780 and a slight increase were seen at rs9265664 (Figure 2c). Like HLA-B, at SNPs, rs9866959 and rs7636790, located upstream and downstream of the CNV region, intensities were almost the same. Within the CNV regions, lower intensities were observed significantly in individuals with above CNV (Figure 2d).

### Validation of CNV Calls Using Quantitative PCR

Among the top candidates, the gene containing loci (MAP4K3, HLA-B and EPHA3), which were validated by LRR values were further subjected to TaqMan CNV detection analysis. The PennCNV results of MAP4K3 and HLA-B, were not validated and thus concluded as false positives. For MAP4K3, the cases shown to have one extra copy by PennCNV, were found to have two copies of the CNV. On the other hand, one control sample which was shown to have two copies by PennCNV, had one copy of the CNV in the MAP4K3 locus (Figure S3a). Similarly, HLA-B results were not validated in eight ALS patients, found to have deletions according to PennCNV, however, heterozygous deletions were found in other controls and ALS patients who were shown to have two copies by PennCNV (Figure S3b).As opposed to the above results, for the EPHA3 locus, two ALS patients and 11 healthy controls with deletions were validated by TaqMan CNV assay. All other individuals seven ALS and seven controls harboring two copies according to the PennCNV results, were found to be in accordance (Figure S3c).

## Discussion

In this study, we have carried out, for the first time, a CNV analysis in a Turkish ALS cohort of 117 ALS patients and 109 controls (GWAS data available upon request). After validation analyses, EPHA3 was shown to be a potential protective factor for ALS.

Common and rare genetic variants like SNPs and CNVs may contribute to complex disease development. Candidate SNPs in several genes, including DPP6, ITPR2, KIFAP3 and UNC13A, were previously shown to be associated with ALS using the GWAS platform although the contribution of these SNPs to disease pathogenesis remained questionable in different studies [18], [19], [20], [21]. The more recently discovered CNVs, on the other hand, are also abundant and dynamic throughout the genome and they can cause genetic variations even between two closely related individuals. Because of their much larger sizes, CNVs may have more drastic effects on the human genome, thus on complex disease development in humans. [22].

Our findings are not in complete accordance with similar studies performed in European and US-Eurepean populations; this may be on one hand due to our relatively small sample size and on the other hand due to the general discordance between GWAS/CNV studies performed so far. Very importantly, the differences may also be population-specific. Considering the great ethnic heterogeneity of the Turkish population, to confirm our findings, CNV analysis has to be expanded to a larger and independent Turkish cohort; a very well-characterized and well-matched cohort is required for unbiased results in a heterogeneous population.

Rare CNVs have been observed in some patients and controls, including several gene regions, e.g. ACYP2, LPHN3, CSMD1, RTEL1, TAC1, SULF1 and APBA1. None of them has been associated with ALS before. In this study, the CNVs in the ACYP2 and LPHN3 gene regions are novel and they were not found in our control populations, thus would be classified as promising risk-conferring candidates. The remaining CNVs were all reported in DGV. Four ALS patients in the study cohort had a novel CNV deletion in heterozygous form in the ACYP2 gene. This enzyme family which acts as a phosphatase, serves particularly to modulate Ca^+2^ from the endoplasmic reticulum; like ITPR2 shown to be a risk factor in ALS [20], [23]. All of our ALS patients had a relatively early age of onset (32–44), three with limb and one with bulbar initiation. When statistical analysis was applied, rare CNVs, including the most promising ACYP2, were found to be non-significant, as expected, but this does not conclude that their effects are not considerable, only their presence is limited. Their loss cannot be pathogenic and would not cause toxicity, however this loss may protect cell from stress. To understand rare and novel CNVs and their contributions to complex disease development, further investigation of these CNVs in terms of their functions in the cell, their involvement in cellular pathways and their association with other diseases are necessary.

The most significant CNV, conferring highly increased risk to ALS in our cohort, is the previously described 41 kb-long centromeric heterozygous deletion at the 11p11.12 locus [17]. Although there are no coding genes detected in proximal regions of this candidate locus, there are several transcription factor binding sites. Presence of deletions or duplications in this region may alter arrangements of chromosomal and centromeric parts in this locus. Furthermore, deletions found at 11p11.12 have been implicated in several cancer types and also in the Potocki-Shaffer syndrome [24], [25], [26]. The risk-conferring nature of this locus has to be validated by further genotyping assays.

MAP4K3 has multiple functions in signal transduction of mammalian cells, such as activation of the JNK pathway and regulation of TORC1 signaling. Increased synthesis of the protein due to duplication may result in excess protein through the TORC1 pathway, leading to ER stress and misfolded protein response which are important mechanisms of ALS pathology. PennCNV found a novel significant duplication at the MAP4K3 locus and also intensities (LRR) for MAP4K3 were significant (Figure 2), however, TaqMan CNV genotyping did not confirm the PennCNV results in this cohort. CNVs at HLA-B locus have been reported previously [15], [17] and many HLA genes were found to be associated with several diseases, such as multiple sclerosis [27], [28]. A homozygous deletion at the HLA-B locus found by PennCNV failed to be validated after qPCR. One reason for this failure may be the highly polymorphic structure of HLAs in humans, possibly resulting in different outcomes in GWAS. Upon the results of TaqMan genotyping, the CNVs detected by PennCNV in MAP4K3 and HLA-B were classified as false-positives.

Another candidate gene detected by PennCNV analysis was Epha3, shown to be a protective factor for ALS. Most importantly, validation analysis by qPCR confirmed this result. Ephrin (Eph) receptors are the largest known protein subfamily of receptor tyrosine kinases. This protein family consists of 14 members in A and B subgroups. Ephrin receptors and ligands enable cell to cell interactions. Their signaling also regulates processes during embryonic development, such as neuronal cell migration, vasculogenesis and axon guidance [29]. We observed that deletion of one copy of EPHA3 is significantly higher in controls as compared to ALS patients (p = 0.0065024). In line with this result, in 2012, van Hoecke *et* *al.* reported that EPHA4, one of Ephs, to be a disease-modifier of ALS. Loss of function mutation and knock-down of EPHA4 gene in mutant SOD1 phenotype rescues and enables long survival [30], indicating a protective effect of EPHA4 gene on disease progression. This indicates that Ephs can be protective targets in ALS for therapeutic intervention.

ALS is a complex neurodegenerative disease, with both upper and lower motor neuron involvement. Although the average age of onset is 50–60 years and the average survival around three years, variability in disease initiation and duration vary tremendously. Even manifestation of the disease in affected family members with the same mutation and in the same gene may be variable from complete to restricted penetrance. Genetic modifying factors are thought to underlie this variability; identification of such modifying pathways is of interest as they may be target for therapeutic interventions. This study represents a sound effort to enlarge our knowledge about ALS risk genes through a genome-wide copy number variation screen in the Turkish population. We hope that its novel findings will contribute to the understanding of the complex pathways leading to neurodegeneration and ALS.

## Supporting Information

Figure S1 **CNVs detected in ALS patients and controls by PennCNV tool were plotted using University of California Santa Cruz (UCSC) Genome Browser (** http://genome.ucsc.edu/). (a). Deletion in centromeric region. Chr11: 50,545,009–50,586,426, (b) Deletion in intergenic region. Chr19: 20,860,930–20,875,787, (c) Duplication in MAP4K3 gene. Chr2: 39,372,016–39,428,488, (d) Homozygous deletion in HLA-B gene. Chr6: 31,389,749–31,393,270 (e) Deletion in EPHA3 gene. Chr3: 89,485,137–89,499,861 (f) Duplication in DPYD gene. Chr1: 97,830,032–97,841,389.
(DOCX)

Figure S2 **Plotting CNV calls of patients and control samples using signal intensities by PennCNV. Each dot represents its Log R Ratio and B Allele Frequency of a SNP. Red indicates SNPs in the CNV site, blue represents SNPs neighboring the CNV region.** (a) ALS-274 and Control-329 have one copy deletion (CN = 1) on chromosome 11, between positions 50.4 Mb and 51.1 Mb. (b) ALS-283 and Control-533 have one copy duplication (CN = 3) on chromosome 1, between positions 97.83 Mb and 97.85 Mb. (c) ALS-51 and ALS-106 have one copy deletion (CN = 1) on chromosome 2, between positions 54.2 Mb and 54.37 Mb.
(DOCX)

Figure S3 **TaqMan CNV Assay results of MAP4K3, HLA-B and EPHA3 genes.** Screen shots taken from the CopyCaller software. (a) Dark Blue samples which were found by PennCNV as candidates to have higher copy number of MAP4K3 gene (n = 3). Light blue samples were supposed to have normal copy number (n = 2). (b) Dark Blue samples which were found by PennCNV as candidates to have no copy number of HLA-B gene (n = 0). Light blue samples were supposed to have normal copy number (n = 2). (c) Dark Blue samples which were found by PennCNV as candidates to have single copy number of EPHA3 gene (n = 1). Light blue samples were supposed to have normal copy number (n = 2). C462 and C672 control samples were supposed to have single copy numbers, however, they had no copy numbers of EPHA3 (n = 0).
(DOCX)
