# Supplementary figures and images for: Genome-Wide Copy Number Variation in Sporadic Amyotrophic Lateral Sclerosis in the Turkish Population: Deletion of EPHA3 Is a Possible Protective Factor
Source: PLoS One. 2013 Aug 26;8(8):e72381. doi: 10.1371/journal.pone.0072381 (PMC3753249; doi:10.1371/journal.pone.0072381)

**Figure S1**


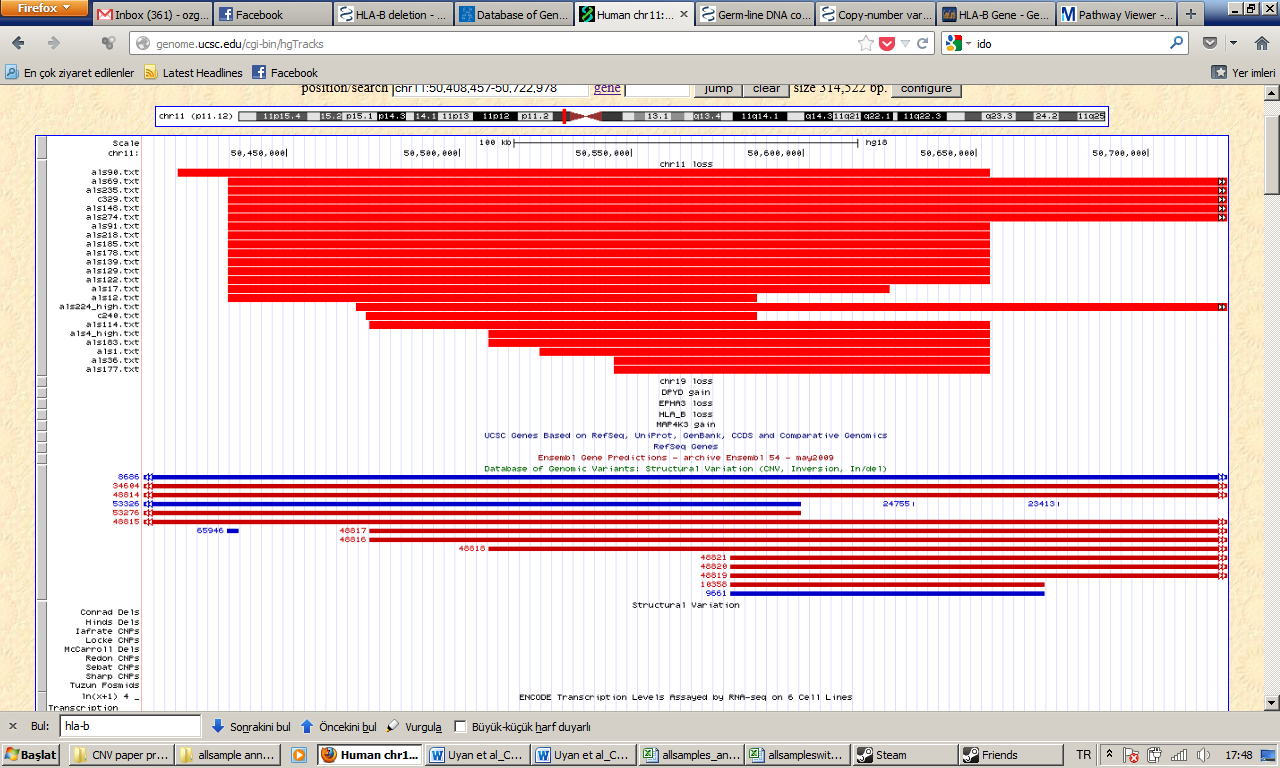


**Figure S1a.**


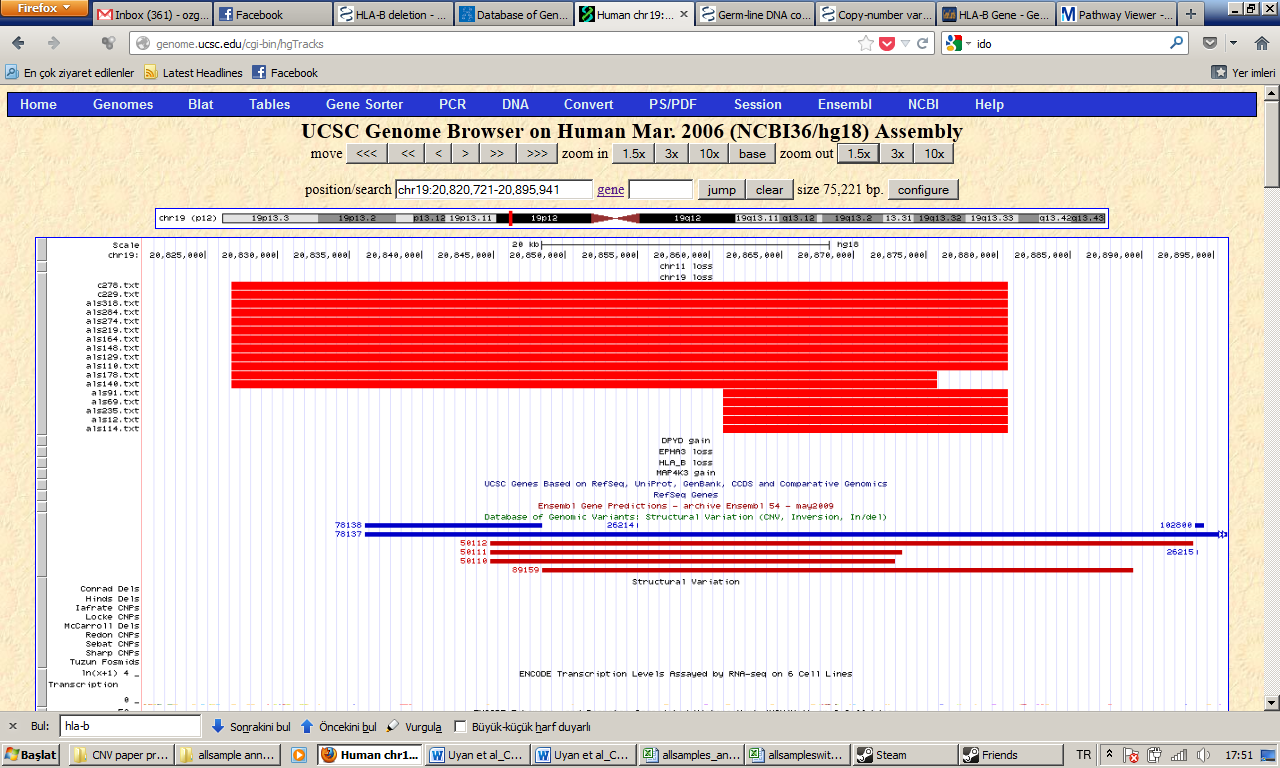


**Figure S1b.**


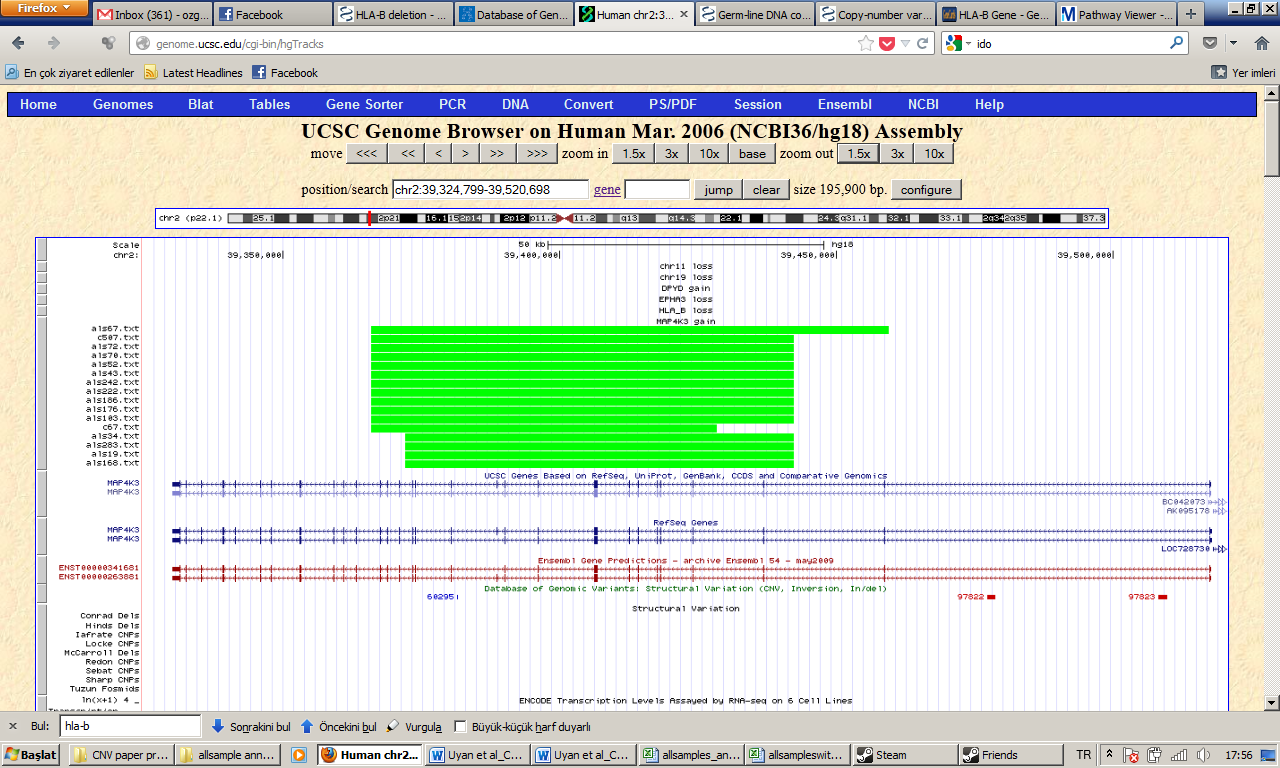


**Figure S1c.**


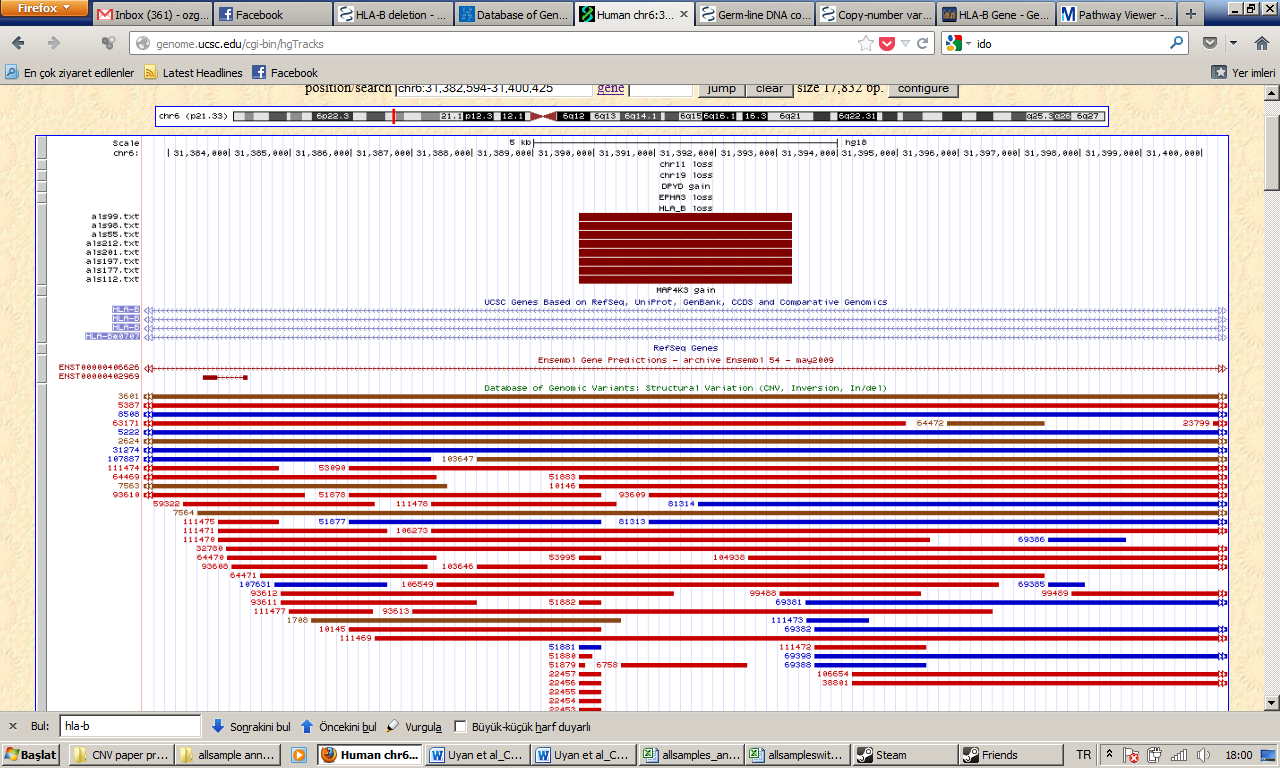


**Figure S1d.**


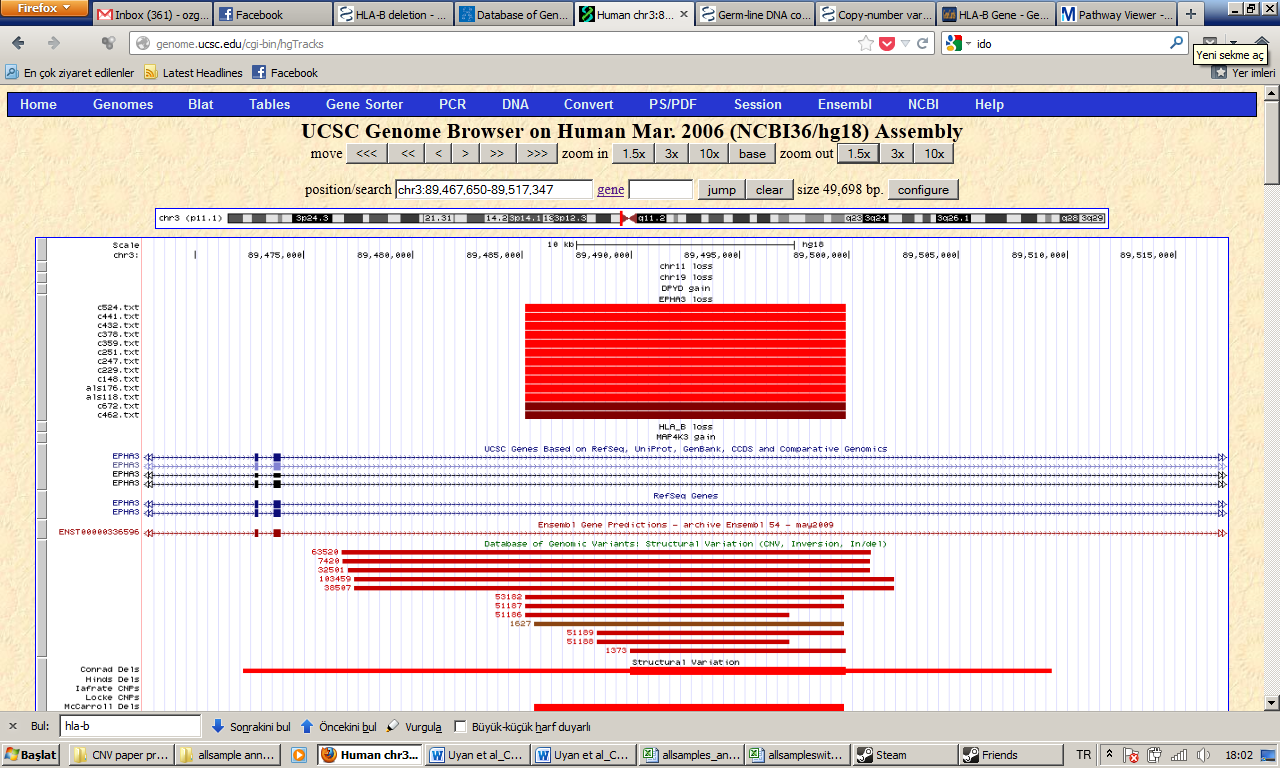


**Figure S1e.**


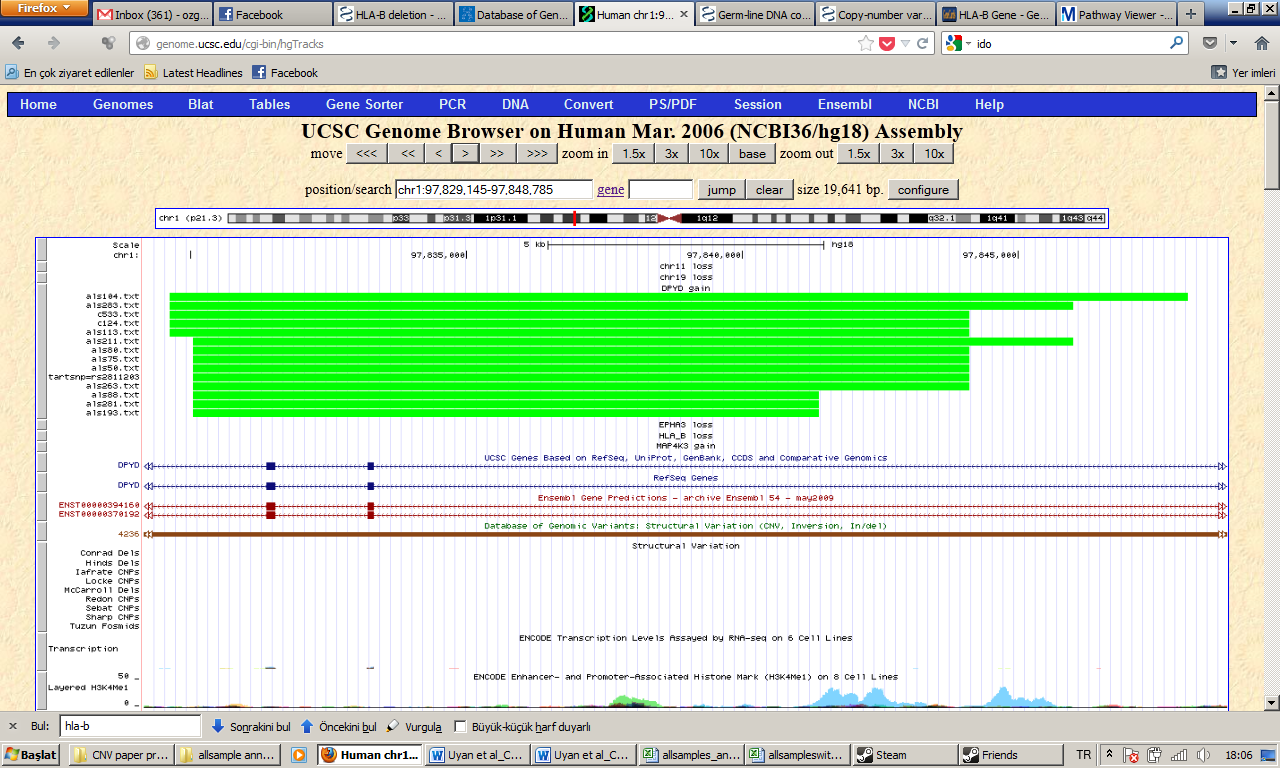


**Figure S1f.**

Supplement: Figure S1 — CNVs detected in ALS patients and controls by PennCNV tool were plotted using University of California Santa Cruz (UCSC) Genome Browser ( http://genome.ucsc.edu/). (a). Deletion in centromeric region. Chr11: 50,545,009–50,586,426, (b) Deletion in intergenic region. Chr19: 20,860,930–20,875,787, (c) Duplication in MAP4K3 gene. Chr2: 39,372,016–39,428,488, (d) Homozygous deletion in HLA-B gene. Chr6: 31,389,749–31,393,270 (e) Deletion in EPHA3 gene. Chr3: 89,485,137–89,499,861 (f) Duplication in DPYD gene. Chr1: 97,830,032–97,841,389. (DOCX) [file pone.0072381.s001.docx]

**Figure S2**


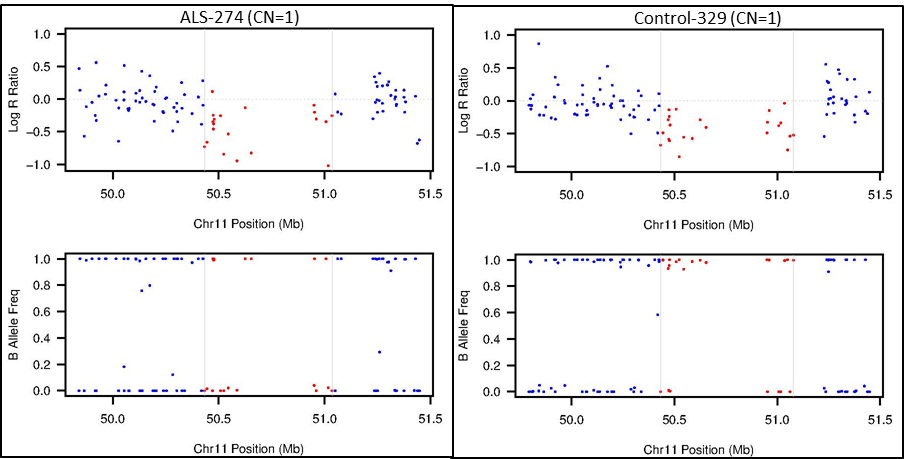


**Figure S2a.**


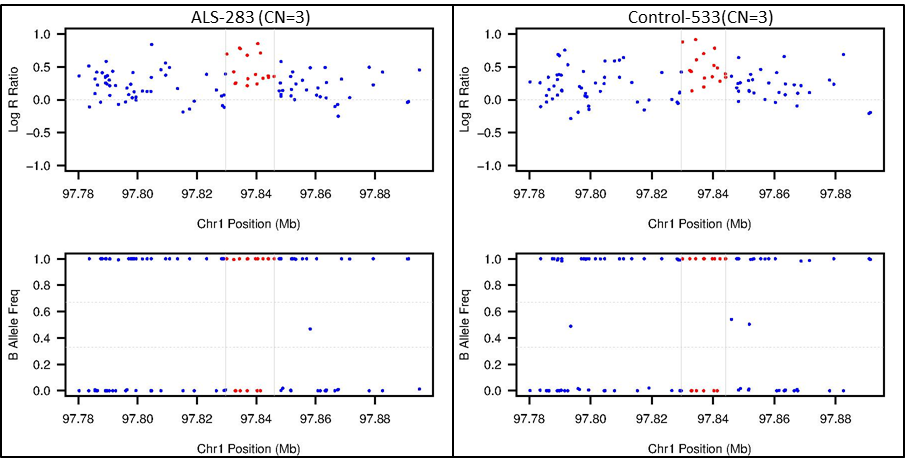


**Figure S2b.**


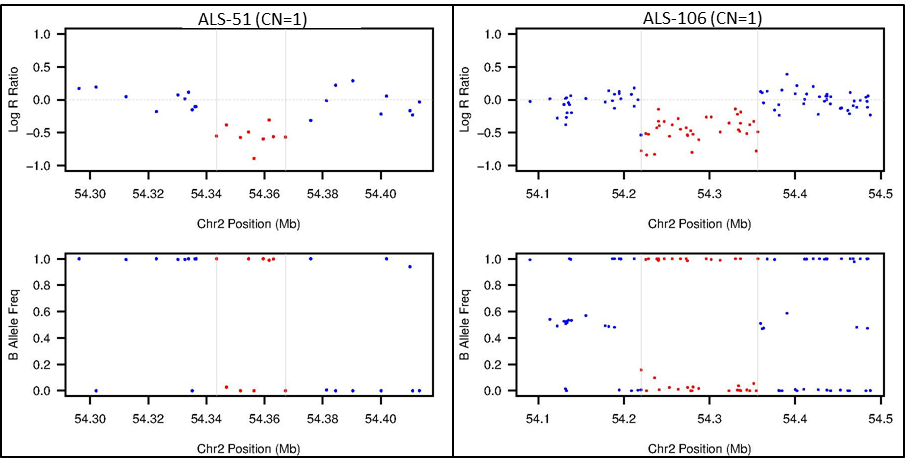


**Figure S2c.**

Supplement: Figure S2 — Plotting CNV calls of patients and control samples using signal intensities by PennCNV. Each dot represents its Log R Ratio and B Allele Frequency of a SNP. Red indicates SNPs in the CNV site, blue represents SNPs neighboring the CNV region. (a) ALS-274 and Control-329 have one copy deletion (CN = 1) on chromosome 11, between positions 50.4 Mb and 51.1 Mb. (b) ALS-283 and Control-533 have one copy duplication (CN = 3) on chromosome 1, between positions 97.83 Mb and 97.85 Mb. (c) ALS-51 and ALS-106 have one copy deletion (CN = 1) on chromosome 2, between positions 54.2 Mb and 54.37 Mb. (DOCX) [file pone.0072381.s002.docx]

**Figure S3:**


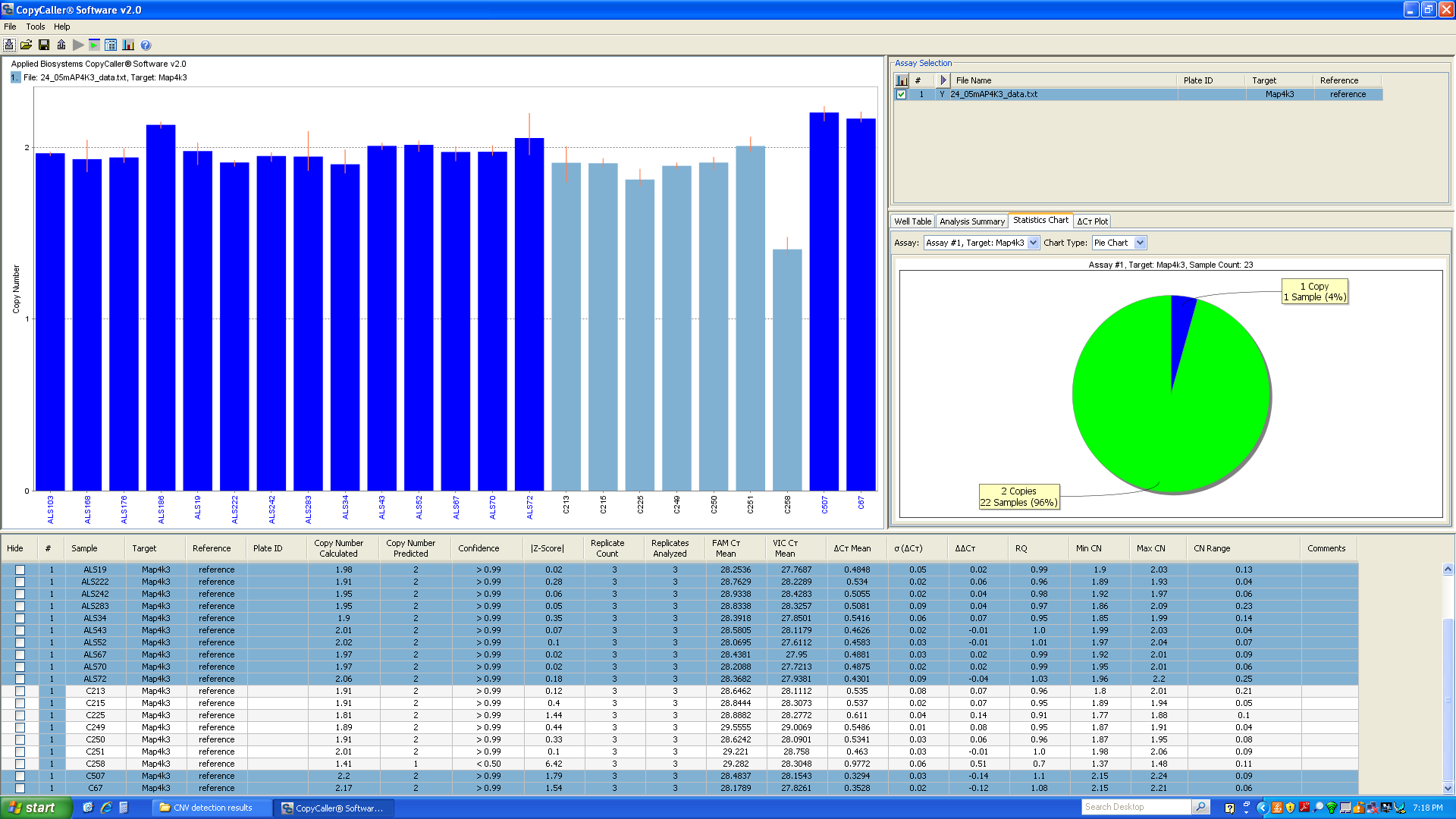


**Figure S3a.**


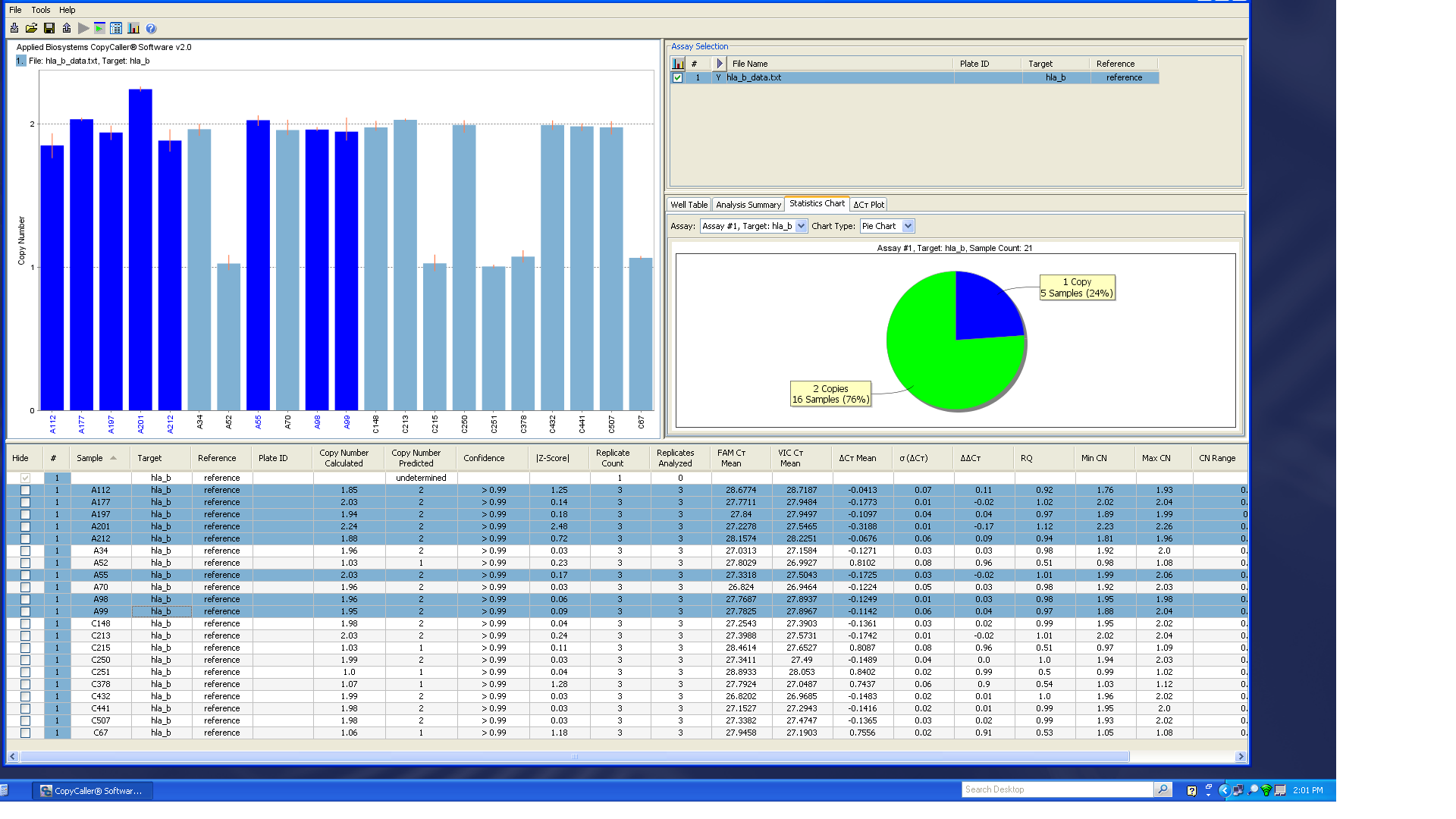


**Figure S3b.**


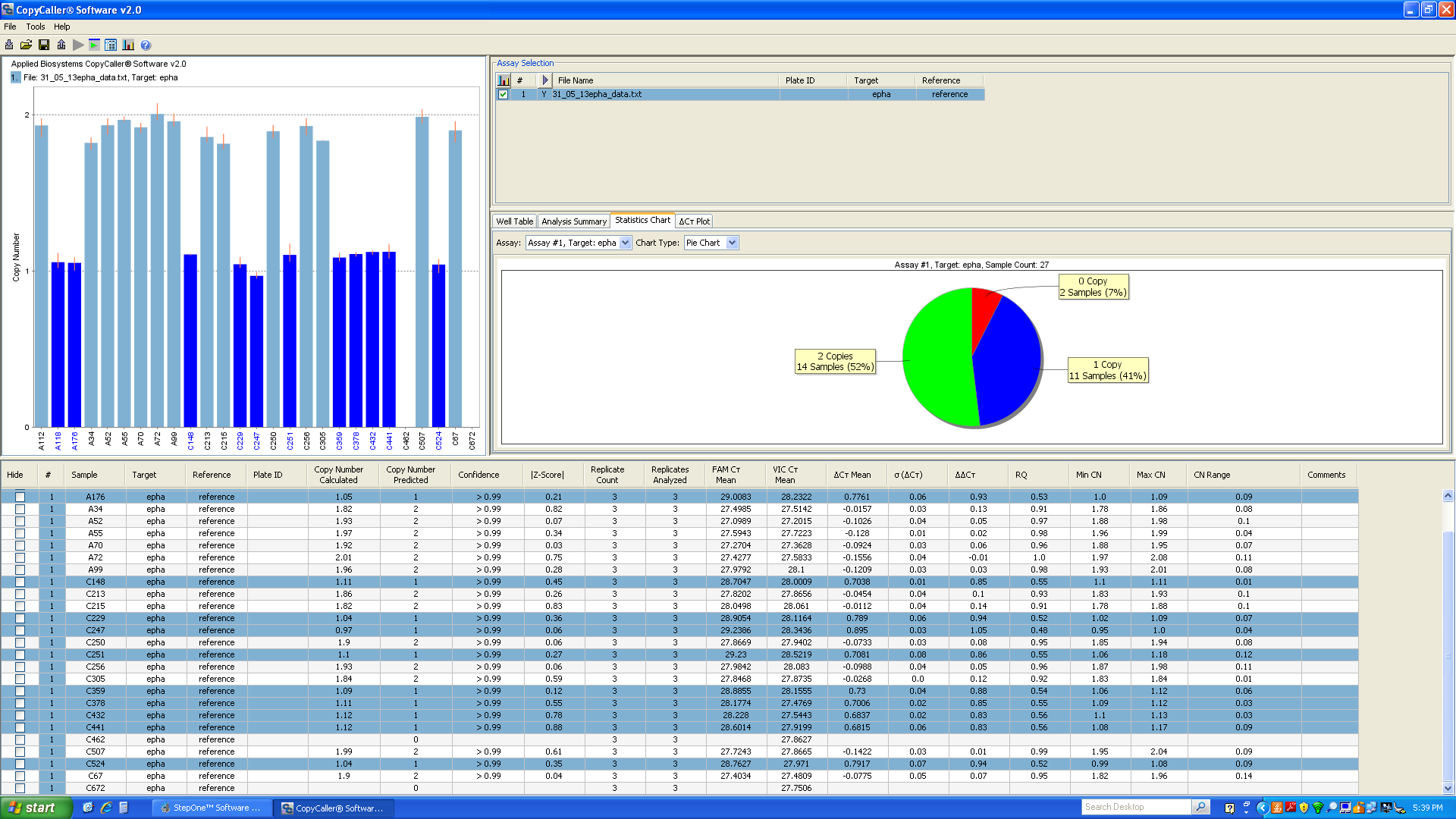


**Figure S3c.**

Supplement: Figure S3 — TaqMan CNV Assay results of MAP4K3, HLA-B and EPHA3 genes. Screen shots taken from the CopyCaller software. (a) Dark Blue samples which were found by PennCNV as candidates to have higher copy number of MAP4K3 gene (n = 3). Light blue samples were supposed to have normal copy number (n = 2). (b) Dark Blue samples which were found by PennCNV as candidates to have no copy number of HLA-B gene (n = 0). Light blue samples were supposed to have normal copy number (n = 2). (c) Dark Blue samples which were found by PennCNV as candidates to have single copy number of EPHA3 gene (n = 1). Light blue samples were supposed to have normal copy number (n = 2). C462 and C672 control samples were supposed to have single copy numbers, however, they had no copy numbers of EPHA3 (n = 0). (DOCX) [file pone.0072381.s003.docx]
